# Patient‐based prediction algorithm of relapse after allo‐HSCT for acute Leukemia and its usefulness in the decision‐making process using a machine learning approach

**DOI:** 10.1002/cam4.2401

**Published:** 2019-07-15

**Authors:** Kyoko Fuse, Shun Uemura, Suguru Tamura, Tatsuya Suwabe, Takayuki Katagiri, Tomoyuki Tanaka, Takashi Ushiki, Yasuhiko Shibasaki, Naoko Sato, Toshio Yano, Takashi Kuroha, Shigeo Hashimoto, Tatsuo Furukawa, Miwako Narita, Hirohito Sone, Masayoshi Masuko

**Affiliations:** ^1^ Faculty of Medicine, Department of Hematology, Endocrinology and Metabolism Niigata University Niigata Japan; ^2^ Department of Hematopoietic Cell Transplantation Niigata University Medical and Dental Hospital Niigata Japan; ^3^ Department of Hematology Nagaoka Red Cross Hospital Nagaoka Japan; ^4^ Laboratory of Hematology and Oncology, Graduate School of Health Sciences Niigata University Niigata Japan

**Keywords:** acute leukemia, allogeneic hematopoietic stem cell transplantation, machine learning, patient‐based prediction, relapse posttransplantation

## Abstract

Although allogeneic hematopoietic stem cell transplantation (allo‐HSCT) is a curative therapy for high‐risk acute leukemia (AL), some patients still relapse. Since patients simultaneously have many prognostic factors, difficulties are associated with the construction of a patient‐based prediction algorithm of relapse. The alternating decision tree (ADTree) is a successful classification method that combines decision trees with the predictive accuracy of boosting. It is a component of machine learning (ML) and has the capacity to simultaneously analyze multiple factors. Using ADTree, we attempted to construct a prediction model of leukemia relapse within 1 year of transplantation. With the model of training data (n = 148), prediction accuracy, the AUC of ROC, and the κ‐statistic value were 78.4%, 0.746, and 0.508, respectively. The false positive rate (FPR) of the relapse prediction was as low as 0.134. In an evaluation of the model with validation data (n = 69), prediction accuracy, AUC, and FPR of the relapse prediction were similar at 71.0%, 0.667, and 0.216, respectively. These results suggest that the model is generalized and highly accurate. Furthermore, the output of ADTree may visualize the branch point of treatment. For example, the selection of donor types resulted in different relapse predictions. Therefore, clinicians may change treatment options by referring to the model, thereby improving outcomes. The present results indicate that ML, such as ADTree, will contribute to the decision‐making process in the diversified allo‐HSCT field and be useful for preventing the relapse of leukemia.

## 1 INTRODUCTION

Allogeneic hematopoietic stem cell transplantation (allo‐HSCT) is an established therapy that is associated with a high rate of curability for acute leukemia (AL).^1^, ^2^, ^3^ However, many patients still relapse after allo‐HSCT, with common causes of death being relapse and leukemia‐associated complications.^3^, ^4^ Since salvage therapy is limited for these patients, their prognosis is very poor, with a probability of long‐term survival of <20%.^5^ Thus, the establishment of prevention strategies against relapse after allo‐HSCT is strongly needed.

Several pretransplant factors that may predict relapse after allo‐HSCT were previously identified, such as patient backgrounds, including age,^6^ the Refined Disease Risk Index (rDRI),^7^ cytogenetic risk,^8^ and the Hematopoietic Cell Transplantation‐Comorbidity Index (HCT‐CI).^9^ Other technical components of allo‐HSCT, including conditioning regimens,^10^, ^11^ the selection of graft sources,^3^, ^12^, ^13^ HLA discrepancies,^14^, ^15^ and other components,^16^, ^17^ are associated with relapse after allo‐HSCT. These prognostic factors have been evaluated with conventional statistical techniques, such as univariate and multivariate analyses, which are model (hypothesis)‐driven techniques; they start with a model and assess whether the data fit the suggested model.^18^ Although these techniques are popular and widely used in the analysis of medical records, they cannot simultaneously process multiple factors. Therefore, the complex network of multiple factors in a patient makes the patient‐based prediction of relapse, which is generally useful in the bedside decision‐making process regarding an indication for or the protocol of allo‐HSCT, difficult.

The application of artificial intelligence (AI) to medicine, particularly machine learning (ML), a type of AI, has recently been attracting increasing attention. Multiple factors may be simultaneously analyzed, and AI may be applied to the examination of complex medical records. Since ML has the capacity to analyze multiple factors, we herein attempted to generate robust and accurate prediction models of relapse after allo‐HSCT, which may be a useful tool in the bedside decision‐making process to select a transplant method for reducing the relapse of leukemia.

## 2 METHODS

### 2.1 Patients

This analysis was a retrospective, data mining, and supervised learning study that included 217 AL patients. They underwent first allo‐HSCT for AL at Niigata University Hospital (n = 148) and Nagaoka Red Cross Hospital (n = 69) between 1990 and 2016 and survived for more than 1 month after transplantation. The median follow‐up of patients was 28.9 months (range 1.2‐223.2 months). The diagnosis and classification of AL were based on criteria according to the WHO classification.^19^, ^20^ Among 217 patients, 135 had acute myeloid leukemia (AML) and 82 had acute lymphoblastic leukemia (ALL). The median age of patients at allo‐HSCT was 38 years (range 16‐67 years old). To compare the risk of relapse, patients were stratified based on rDRI.^7^ (The definitions of rDRI and cytogenetic risk have been excerpted from reference No. 7 in Table S1.) According to rDRI, 14 (6.5%), 121 (55.8%), 51 (23.5%), and 31 (14.3%) patients were at low (LOW), intermediate (INT), high (HI), and very high risk (VH), respectively. Donors were related for 97 patients (44.7%) and were unrelated for 120 (55.3%). Graft sources were peripheral blood stem cells (PBSC) for 47 patients (21.7%, including PBSCs from 22 haploidentical donors), bone marrow (BM) for 123 (56.7%), and cord blood (CB) for 47 (21.7%). Myeloablative conditioning was used for 169 patients (77.9%) and with reduced intensity for 48 (22.1%). Among 22 patients with haploidentical donor graft, thymoglobulin in 14 and post cyclophosphamide in eight patients were used as conditioning. None of the patients received T‐cell–depleted grafts in the present study. The HCT‐CI score was low (0, 1, 2) for 183 (84.3%) patients and high (≥3) for 34 (15.7%) (Detailed information is shown in Table 1).

**Table 1:** Patient characteristics

			Hospital		
Factor		All	Niigata (training set)	Nagaoka (validation set)	
	Number of patients	N = 217	N = 148	N = 69	*P*‐value
Age (range)		38 y (10‐67)	38 y (10‐66)	39 y (14‐67)	0.196
Age (%)	<40 y	119 (54.8)	83 (56.1)	36 (52.2)	
	≤40 y	98 (45.2)	65 (43.9)	33 (47.8)	0.661
Sex (%)	Male	111 (51.2)	74 (50.0)	37 (53.6)	0.663
	Female	106 (48.8)	74 (50.0)	32 (46.4)	
Diagnosis (%)	AML	135 (62.2)	97 (65.5)	38 (55.1)	0.176
	ALL	82 (37.8)	51 (34.5)	31 (44.9)	
	with (9;22)	31 (37.8)	18 (35.3)	13 (41.9)	0.64
Hospital (%)	Niigata	148 (68.2)			
	Nagaoka	69 (31.8)			
rDRI (%)	LOW	14 (6.5)	10 (6.8)	4 (5.8)	0.574
	INT	121 (55.8)	78 (52.7)	43 (62.3)	
	HI	51 (23.5)	36 (24.3)	15 (21.7)	
	VH	31 (14.3)	24 (16.2)	7 (10.1)	
Graft source (%)	BM	123 (56.7)	85 (57.4)	38 (55.1)	0.115
	PBSC	25 (11.5)	19 (12.8)	6 (8.7)	
	HAPLO‐PBSC	22 (10.1)	18 (12.2)	4 (5.8)	
	CB	47 (21.7)	26 (17.6)	21 (30.4)	
Donor type (%)	Unrelated	120 (55.3)	77 (52.0)	43 (62.3)	0.187
	Related	97 (44.7)	71 (48.0)	26 (37.7)	
Conditioning (%) including	MAC	169 (77.9)	110 (74.3)	59 (85.5)	0.079
	RIC	48 (22.1)	38 (25.7)	10 (14.5)	
	Thymoglobulin	14 (6.5)	13 (8.8)	1 (1.4)	
	Post cyclophosphamide	8 (3.7)	5 (3.4)	3 (4.3)	
HCT_CI score (%)	≤2	183 (84.3)	126 (85.1)	57 (82.6)	0.69
	≤3	34 (15.7)	22 (14.9)	12 (17.4)	
NRM (%)	Yes	42 (19.4)	29 (19.6)	13 (18.8)	1
Using TBI (%)	No	19 (8.8)	17 (11.5)	2 (2.9)	0.041
	Yes	197 (91.2)	131 (88.5)	66 (97.1)	
Relapse within 1 y (%)	Yes	69 (31.8)	51 (34.5)	18 (26.1)	0.273
OS	Months (range)	28.9 (1.2‐223.2)	31.4 (1.2‐223.2)	27.3 (1.2‐127.1)	0.382
RFS	Months (range)	20.6 (1.0‐223.2)	20.4 (1.0‐223.2)	20.7 (1.0‐124.8)	0.816

No significant differences were observed between the training and validation sets.

Abbreviations: ALL, acute lymphoblastic leukemia; AML, acute myeloid leukemia; BM, bone marrow; CB, cord blood; HAPLO‐PBSC, PBSC from haploidentical donors; HCT‐CI, Hematopoietic Cell Transplantation‐Comorbidity Index; HI, high risk; INT, intermediate risk; LOW, low risk; MAC, myeloablative conditioning; NRM, nonrelapse mortality; OS, overall survival; PBSC, peripheral blood stem cells; rDRI, the Refined Disease Risk Index; RFS, relapse‐free survival; RIC, reduced intensity conditioning; TBI, total body irradiation; VH, very high risk.

The present study was performed in accordance with the Japanese Ethical Guidelines for Medical and Health Research Involving Humans and approved by the Ethical Committee of our facilities.

### 2.2 ML and ADTree

The alternating decision tree (ADTree), one component of the ML approach based on AI, is a successful classification method. ADTree combines decision trees with the predictive accuracy of boosting into a set of interpretable classification rules. Boosting influences the node‐weighted score (NW) by repeating the sample classification with each node and calculating errors and classification confidence each time. Moreover, it repeats re‐weighting the training samples to focus on the most problematic factor.^21^ (A more detailed principle is in reference No. 21).

Since ADTree learns previous data and predicts future classifications or discriminations, we used this algorithm in the present study as ML. ADTree was performed using WEKA software (Ver.3.9.1, Machine Learning Group at the University of Waikato, New Zealand, https://www.cs.waikato.ac.nz/ml/weka/index.html). The algorithm model was trained and tested using 10‐fold cross‐validation on the training data set (Niigata group) and validated again on the validation data set (Nagaoka group). The model evaluated the prediction accuracy and area under the curve (AUC) of the receiver operating characteristic (ROC) analysis, which discriminates the true prediction rate from a false prediction rate (FPR, also called the specificity). The tree was analyzed with the number of nodes between 6 and 11 and we adopted the number of nodes showing the highest κ‐statistic value (Table S2).

### 2.3 Other statistical analyses

Group comparisons for continuous or categorical variables were evaluated using the Mann‐Whitney *U* test or Fisher's test. All times to events were computed from the date of transplantation. Overall survival (OS) was analyzed as the time until death or lost to the follow‐up with the Kaplan‐Meier estimator. The cumulative incidence of relapse (CIR) was assessed as the time of hematological relapse. In the case of no remission after allo‐HSCT, the time of relapse was defined when blasts circulating in peripheral blood or BM were detected. The cut‐off value for age used in adjustments in the multivariate analysis was set to 40 years.^6^ Univariate and multivariate analyses for CIR were performed using Fine and Gray models. Apart from ADTree, statistical analyses were performed using R‐statistical software version 3.4.3 (The R Foundation for Statistical Computing) and EZR (Saitama Medical Center, Jichi Medical University), which is a graphical user interface for R.^22^ The significance of differences was considered to be *P* < 0.05 with a two‐sided test.

## 3 RESULTS

### 3.1 Outcomes

**Figure 1:**
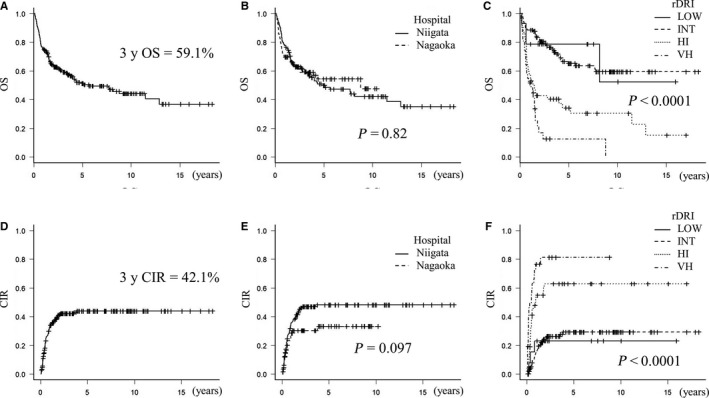
OS and CIR of all patients. (A) and (D); OS and CIR of all patients. One‐ and 3‐year OS rates were 75.1% (95% CI: 68.8%‐80.3%) and 59.1% (95% CI: 52.0%‐65.6%), respectively. (B) and (E); No significant differences were observed in OS (*P* = 0.82) or CIR (*P* = 0.097) between Niigata Hospital (training set) and Nagaoka Hospital (validation set). (C) and (F) When stratified based on rDRI, OS (*P* < 0.0001) and CIR (*P* < 0.0001) showed significant differences among the categories

One‐ and 3‐year OS rates were 75.1% (95% CI: 68.8%‐80.3%) and 59.1% (95% CI: 52.0%‐65.6%), respectively (Figure 1A). One‐ and 3‐year CIR rates were 33.8% (95% CI: 26.9%‐40.0%) and 42.1% (95% CI: 34.7%‐48.6%), respectively (Figure 1D). Most cases of relapse occurred within 1 year of allo‐HSCT (Table 2 **, **Figure 1D,1,1). There were six cases of no remission (2.8%) after allo‐HSCT. Nonrelapse mortality (NRM) was 19.4% (n = 42) (Detailed information on NRM is shown in Table S3).

**Table 2:** Patient outcomes for OS and CIR

			OS (%) (95% CI)			CIR (%) (95% CI)		
			1 y	3 y	*P*‐value	1 y	3 y	*P*‐value
	All	N = 217	75.1 (68.8‐80.3)	59.1 (52.0‐65.6)		33.8 (26.9‐40.0)	42.1 (34.7‐48.6)	
Age	<40 y	N = 119	75.6 (66.9‐82.4)	59.9 (50.3‐68.3)	*P* = 0.714	34.3 (24.9‐42.5)	39.9 (28.5‐49.4)	*P* = 0.532
	≤40 y	N = 98	74.5 (64.6‐82.0)	58.2 (47.3‐67.6)		33.2 (22.7‐42.3)	39.9 (28.5‐52.5)	
Sex	Male	N = 111	72.1 (62.7‐79.5)	54.7 (44.7‐63.5)	*P* = 0.123	36.9 (26.8‐45.6)	43.9 (33.1‐52.9)	*P* = 0.051
	Female	N = 106	78.3 (69.2‐85.0)	63.8 (53.3‐72.5)		30.7 (21.0‐39.1)	40.4 (29.8‐49.3)	
Diagnosis	AML	N = 135	73.3 (65.0‐80.0)	57.4 (48.3‐65.5)	*P* = 0.457	36.1 (27.2‐44.0)	43.1 (33.6‐51.2)	*P* = 0.410
	ALL	N = 82	78.0 (67.4‐85.6)	61.9 (67.4‐85.6)		29.9 (18.8‐39.4)	40.4 (27.9‐50.7)	
Hospital	Niigata (training data)	N = 148	77.7 (70.1‐83.6)	59.3 (50.6‐66.9)	*P* = 0.82	36.0 (22.7‐27.6)	46.9 (37.8‐54.7)	*P* = 0.097
	Nagaoka (validation data)	N = 69	69.6 (57.2‐79.0)	59.1 (46.0‐70.0)		28.5 (16.4‐38.9)	30.3 (17.8‐40.9)	
rDRI	LOW	N = 14	78.6 (47.2‐92.5)	78.6 (47.2‐92.5)	***P* < 0.0001**	23.1 (0.0‐42.9)	NA	***P* < 0.0001**
	INT	N = 121	88.4 (81.2‐93.0)	76.3 (67.2‐83.1)		15.8 (0.6‐8.8)	26.3 (1.5‐17.5)	
	HI	N = 51	56.9 (42.2‐69.1)	40.0 (26.8‐53.6)		54.9 (9.0‐37.6)	62.8 (12.5‐45.0)	
	VH	N = 31	51.6 (33.0‐67.4)	12.6 (3.3‐28.3)		76.5 (25.2‐55.1)	81.2 (32.4‐58.9)	
Graft	BMT	N = 123	78.9 (70.5‐85.1)	67.0 (57.8‐74.6)	***P* = 0.00687**	29.9 (21.0‐37.7)	37.3 (27.7‐45.6)	***P* = 0.005**
	PB	N = 25	64.0 (42.2‐79.4)	44.3 (23.4‐63.4)		51.6 (26.2‐68.2)	61.5 (34.5‐77.3)	
	HAPLO‐PB	N = 22	63.6 (40.3‐79.9)	33.5 (14.6‐53.7)		56.1 (28.9‐73.0)	69.3 (38.0‐84.8)	
	CBT	N = 47	76.6 (61.7‐86.3)	56.3 (38.9‐70.5)		23.6 (9.6‐35.4)	31.7 (15.7‐44.7)	
Graft	Related	N = 97	73.2 (63.2‐80.9)	56.4 (45.6‐65.8)	*P* = 0.569	40.9 (30.0‐50.2)	51.9 (40.2‐61.2)	***P* = 0.014**
	Unrelated	N = 120	76.7 (68.‐83.3)	61.4 (51.6‐69.7)		27.8 (18.9‐35.7)	33.8 (24.3‐42.2)	
Conditioning	MAC	N = 169	74.6 (67.3‐80.5)	60.8 (52.8‐67.9)	*P* = 0.598	32.8 (25.1‐39.8)	40.6 (32.3‐47.9)	*P* = 0.492
	RIC	N = 48	77.1 (62.5‐85.6)	52.9 (36.8‐66.6)		37.1 (21.3‐49.7)	47.1 (29.9‐60.1)	
Using TBI	No	N = 19	68.4 (42.8‐84.4)	45.6 (22.3‐66.3)	*P* = 0.303	47.4 (19.4‐65.6)	32.6 (28.0‐80.5)	*P* = 0.051
	Yes	N = 197	75.6 (69.0‐81.0)	60.5 (53.0‐72.5)		32.6 (25.5‐39.1)	40.5 (32.8‐47.3)	
HCT_CI score	≤2	N = 183	76.0 (69.1‐81.5)	59.2 (51.4‐66.2)	*P* = 0.777	35.0 (27.5‐41.7)	42.8 (34.8‐49.8)	*P* = 0.526
	≤3	N = 34	70.6 (52.2‐83.0)	58.4 (40.1‐72.9)		27.3 (9.2‐41.8)	38.6 (17.6‐54.2)	

Abbreviations: ALL, acute lymphoblastic leukemia; AML, acute myeloid leukemia; BM, bone marrow; CB, cord blood; CIR, cumulative incidence of relapse; HAPLO‐PBSC, PBSC from haploidentical donors; HCT‐CI, Hematopoietic Cell Transplantation‐Comorbidity Index; HI, high risk; INT, intermediate risk; LOW, low risk; MAC, myeloablative conditioning; OS, overall survival; PBSC, peripheral blood stem cells; rDRI, the Refined Disease Risk Index; RIC, reduced intensity conditioning; TBI, total body irradiation; VH, very high risk.

### 3.2 Univariate and multivariate analyses for CIR

rDRI (*P* < 0.0001), the graft source (*P* = 0.005), and donor type (related or unrelated, *P* = 0.014) were identified as risk factors for relapse, while age (*P* = 0.532), sex (*P* = 0.051), the conditioning regimen (*P* = 0.492), TBI (*P* = 0.051), diagnosis (*P* = 0.410), and HCT‐CI (*P* = 0.526) did not contribute to CIR in this cohort. rDRI (*P* < 0.0001) and the graft source (*P* = 0.00687) influenced OS, whereas the donor type did not (*P* = 0.569) (Table 2, Figure 1).

In the multivariate analysis, rDRI was identified as a risk factor for CIR (*P* < 0.0001), particularly rDRI; VH (HR 6.236, 95% CI: 1.696‐22.93, *P* = 0.006) (Table 3).

**Table 3:** Multivariate analysis of CIR

Factor	Hazard ratio	(95% CI)	*P*‐value
Age < 40 y	0.783	0.453‐1.353	0.380
Conditioning; RIC	0.680	0.336‐1.376	0.280
rDRI			**<0.0001**
Compared to LOW			
DRI—INT	0.877	0.255‐3.026	0.840
DRI—HI	2.953	0.854‐10.210	0.087
DRI—VH	6.236	1.696‐22.930	**0.006**
ALL	1.045	0.628‐1.740	0.860
Graft source			0.993
Compared to BMT			
graft—CBT	0.913	0.435‐1.918	0.810
graft—HAPLO‐PBSC	1.051	0.419‐2.635	0.920
graft—PBSC	0.960	0.413‐2.227	0.920
Donor type: Related	1.401	0.781‐2.514	0.260
Female	1.175	0.714‐1.936	0.530
Using TBI	0.555	0.230‐1.339	0.190

In a multivariate analysis, rDRI was identified as a risk factor for CIR alone (*P* < 0.0001), particularly rDRI; VH (HR 6.236, 95% CI: 1.696‐22.93, *P* = 0.006).

Abbreviations: ALL, acute lymphoblastic leukemia; BM, bone marrow; CB, cord blood; HAPLO‐PBSC, PBSC from haploidentical donors; HI, high risk; INT, intermediate risk; LOW, low risk; PBSC, peripheral blood stem cells; rDRI, the Refined Disease Risk Index; RIC, reduced intensity conditioning; TBI, total body irradiation; VH, very high risk.

### 3.3 The model constructed with ADTree was generalized and highly accurate

Since most cases of relapse occurred within one year in this cohort, the presence or absence of relapse within one year of allo‐HSCT was set as learning content in ADTree to construct a prediction model. We selected seven factors for learning: age, diagnosis, rDRI, donor type, graft, the use of TBI, and the conditioning regimen, which were common prognostic factors, and all were identified prior to transplantation. GVHD and the progression of chimerism, which occur after transplantation, were intentionally excluded as analysis factors.

**Figure 2:**
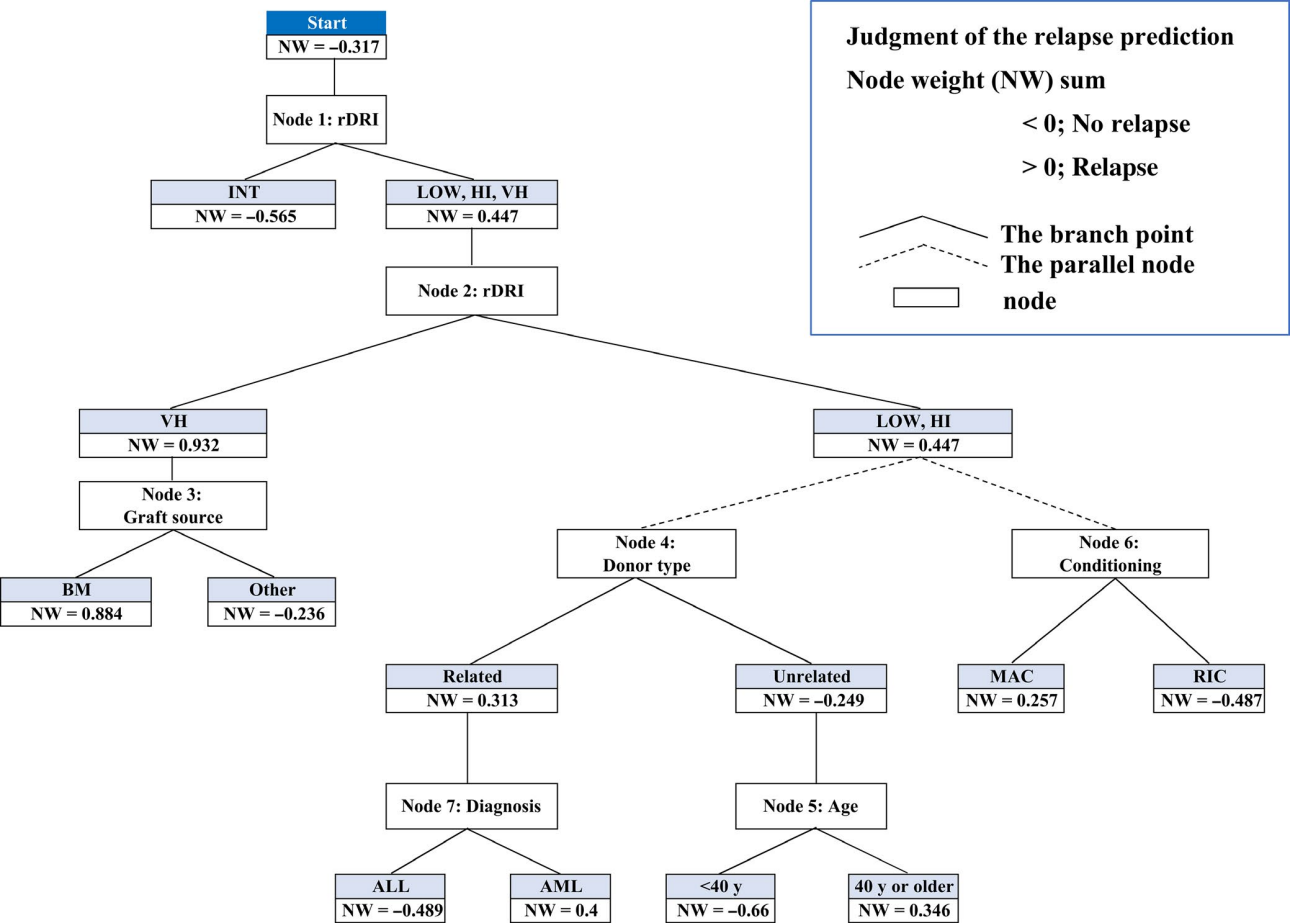
Relapse prediction model; Graphical output. Each score beside nodes showed a prediction node weight (NW); NW < 0 means a lower relapse risk and NW > 0 a higher relapse risk. The final judgment of the AL relapse prediction was achieved by summing all the nodes through which it passed (NW sum). The NW sum > 0 predicted relapse and <0 predicted no relapse in this model

The graphical output of ADTree from the training set (n = 148) is shown in Figure 2. The prediction accuracy, AUC of ROC, and κ‐statistic value of this model were 78.4%, 0.746, and 0.508, respectively. Thirteen out of 97 patients who remained in remission in the first year were predicted to relapse (Detailed results are shown in Table 4); therefore, FPR of the relapse prediction was as low as 0.134. In an evaluation of the model with the validation set (n = 69), the prediction accuracy, AUC of ROC, and FPR of the relapse prediction were similar at 71.0%, 0.667, and 0.216, respectively. These results suggest that the model, constructed with ADTree, was generalized and highly accurate (Table 5, Table S2).

**Table 4:** The actual number of patients and the prediction number of relapse

Training set (n = 148)		Prediction		Total
		No relapse	Relapse	
Actual number	No relapse	84	13	97
	Relapse	19	32	51
	Total	103	45	148

Validation set (n = 69)		Prediction		Total
		No relapse	Relapse	
Actual number	No relapse	40	11	51
	Relapse	9	9	18
	Total	49	20	69

		Prediction	
		No relapse	Relapse
Actual number	No relapse	True negative	False positive
	Relapse	False negative	True positive

**Table 5:** Comparison of predictability

				In relapse			
	Accuracy	AUC	κ‐statistic	TPR	TNR	FPR	FNR
Niigata group (Training set)	78.4%	0.746	0.508	0.627	0.866	0.134	0.373
Nagaoka group (Validation set)	71.0%	0.667	0.274	0.500	0.784	0.216	0.500

In this prediction model, true‐positive rate (TPR, also called the sensitivity) and false‐positive rate (FNR, it shows miss rate) in relapse were not sufficiently. However, true–negative rate (TNR, also called specificity) was high‐ and false‐positive rate (FPR, it means probability of false alarm) in relapse was very low. Therefore, clinicians may consider changes in treatment options if relapse is predicted.

AUC; area under the curve, Accuracy = (true positive + true negative)/ all, TPR = true positive/ (true positive + false negative) = 1‐FNR, FNR = false negative/ (false negative + true positive) = 1‐TPR, TNR = true negative/ (true negative + false positive) = 1‐ FPR, FPR = false positive/ (false positive + true negative) = 1‐TNR.

### 3.4 The branch point of therapeutic options by referring to the model

**Figure 3:**
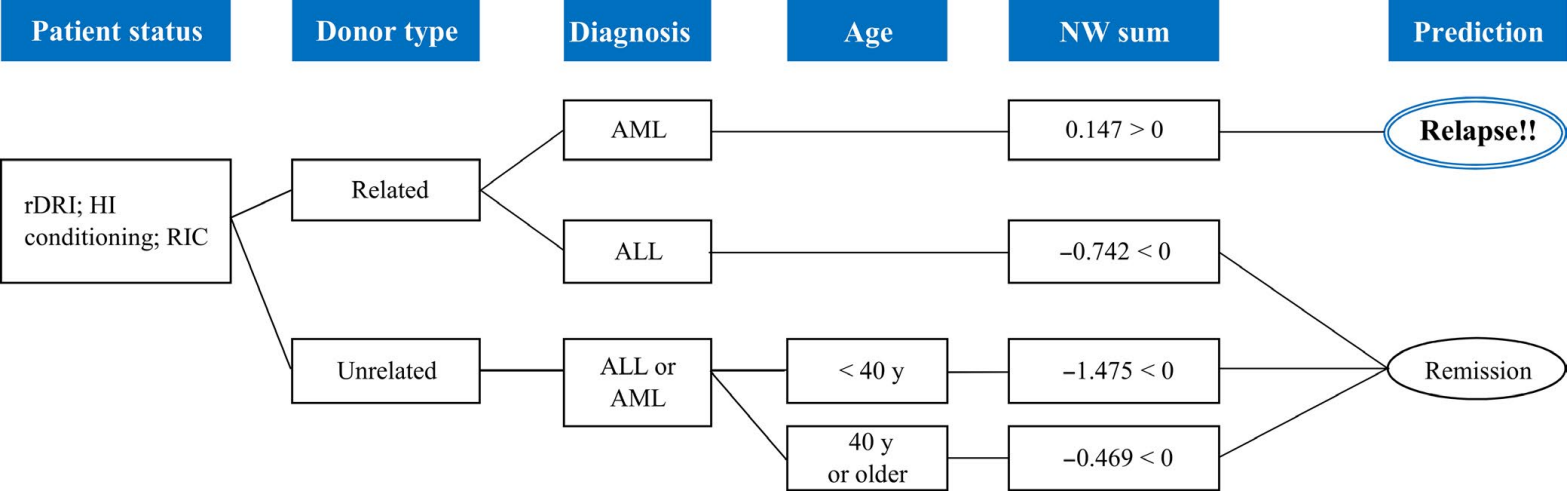
Example of a simulation. In high‐risk AML, the branch point of therapeutic options was the donor type from our simulation

Each score besides that for nodes showed a prediction node weight (NW); NW < 0 means a lower relapse risk and NW > 0 a higher relapse risk. For example, among the same node level, donor type (node 4), an unrelated donor showed a lower risk (NW, −0.249) than a related donor (NW, 0.313), and this result was the same as that in the univariate analysis on CIR. The final judgment of the AL relapse prediction was performed by summing all the nodes through which it passed (NW sum). The NW sum > 0 predicted relapse and < 0 predicted no relapse in this model. According to the model, if ALL patients with rDRI HI receive allo‐HSCT using the RIC regimen and a related donor, the NW sum is −0.742 < 0, which predicts no relapse. Moreover, if the diagnosis is AML, the NW sum is 0.147 > 0, which predicts relapse within 1 year of allo‐HSCT. However, in the case of an unrelated donor, the relapse prediction changes; the NW sum becomes −1.475 (age ≤ 40 years) and −0.469 < 0 (>40 years), indicating no relapse (Figure 3).

## 4 DISCUSSION

Historically, AI and ML were initially developed for image and voice recognition and were subsequently applied to the analysis of data sets of large volumes, such as purchase records.^18^ AI and ML are now expected to handle and analyze complex medical records. When clinical study reports were searched using the following keywords on Pubmed: “machine learning”, “diagnosis”, and “prognosis”, 18 reports in 2000 and 185 in 2010 were hit. Between 2015 and 2018, approximately 1000 reports were searched for each year.

Some groups in the hematology field also attempted to use AI and ML. Shouval et al analyzed the data of approximately 20 000 patients in the European Society for Blood and Marrow Transplantation with ADTree and succeeded in constructing a prediction model of early NRM after allo‐HSCT.^23^ They also evaluated the same data set and compared it with six other ML programs. All programs showed high predictability and versatility.^24^ AI and ML have also been applied in the following fields: the morphological analysis of blood cells,^25^ the identification of prognostic factors of ALL in childhood,^26^ and the differential diagnosis of hematological diseases.^27^

High CIR rates after allo‐HSCT represent a clinical issue that needs to be resolved in adverse risk AL.^3^, ^4^, ^5^ To improve outcomes, attempts are being made to develop strategies that reduce the risk of relapse. Many technical options are now available for allo‐HSCT.^3^, ^10^, ^11^, ^12^, ^13^, ^14^, ^15^, ^16^, ^17^ Furthermore, with the establishment of a safer method for elderly patients,^28^ the number of patients indicated for allo‐HSCT has increased. Since the technique of allo‐HSCT is very diversified and complex, some clinicians may have difficulties selecting treatment options that improve the outcomes of each patient.

The concept of this analysis is “Changing therapy options to avoid leukemia relapse according to model predictions”. We focused on what may be modified factors (such as conditioning or the graft source) and what are fixed factors (including age, disease status, or diagnosis) in the analysis prior to transplantation. Furthermore, a unique point in our analysis was the visualization of the branch point of treatment using ADTree (Figure 2). Although previous studies using AI or ML mainly aimed at discrimination and diagnosis, as described above, we herein attempted to construct patient‐based treatment algorithms by applying AI. In high‐risk AML, the branch point of therapeutic options from our simulation was the donor type (Figure 3). By referring to the prediction results of ADTree, clinicians may change treatment options, thereby improving outcomes. In the present results (Figure 2, at node 1), INT showed NW −0.565, whereas LOW together with HI and VH showed NW 0.447, indicating that LOW was a higher relapse risk than INT, which was unexpected. LOW patients generally do not need to receive allo‐HSCT at first remission. LOW patients who received allo‐HSCT failed first‐line therapy, and may have a worse status than other patients; therefore, we speculated that ADTree judged LOW as a higher relapse risk than INT. This result suggests that ADTree provides us with different information and interactions from existing knowledge.

Medical records contain very diverse information. Patients have different backgrounds that are generally not ideal for statistical analyses. Classical statistical techniques require “noise” to be removed from data when medical records are analyzed. Model (hypothesis)‐driven statistical techniques have identified many prognostic factors, but have been unable to adjust for each patient with individual factors, which are sometimes considered to be “noise”.^18^ These disadvantages have led to difficulties in the construction of “individualized transplantation therapy” for patients in clinical settings. One of the differences between the conventional method and ML is that the former focuses on proving “whether the hypothesis is true”, whereas ML, such as ADTree, “attempts to explain previous data and predict the future”. This difference is expected to be an advantage when using ML for the analysis of medical records.

The limitation of the present study is that the volume of patient data for ADTree to learn was relatively small. The higher the amount of learning, the greater the prediction accuracy, and, thus, it is possible to construct a model that is more useful for the bedside decision‐making process by clinicians. Furthermore, other factors, such as information on chromosome or genetic abnormalities and the chemotherapy protocol, HLA discrepancy rate, and the posttransplant maintenance therapy (donor lymphocyte infusion, azacytidine, et al) are needed to develop ADTree. The planned DLI and targeted posttransplant therapy may be effective at preventing relapse, but this study did not include the patients who received these therapies, so we could not evaluate their effects. The addition of social environments and educational history, which are complex factors, may also be beneficial.^29^ The outcomes of allo‐HSCT may vary among transplant centers. The present results suggest that ADTree is currently applicable to bedside decision‐making in single institutions.

In conclusion, we attempted to generate robust and accurate prediction models of relapse after allo‐HSCT that will contribute to preventing the relapse of leukemia. AI and ML, such as ADTree, may improve the decision‐making process for therapy in the diversified allo‐HSCT field. The usefulness of AI and ML is now being demonstrated, and further clinical applications are expected in the future.

## CONFLICT OF INTEREST

Nothing to report.

## AUTHOR CONTRIBUTIONS

KF designed this study, analyzed the data, and wrote the manuscript. TT, YS, TF, MN, and HS participated in the conception of the study and in providing patient data. MM revised the manuscript and provided administrative support for the study. All authors reviewed and approved the final version of the manuscript.
