# Supplementary material for: Patient‐based prediction algorithm of relapse after allo‐HSCT for acute Leukemia and its usefulness in the decision‐making process using a machine learning approach
Source: Cancer Med. 2019 Jul 15;8(11):5058–67. doi: 10.1002/cam4.2401 (PMC6718546; doi:10.1002/cam4.2401)
Supplement: Supplementary file 1 [file CAM4-8-5058-s001.docx]

**Supplementary Tables:**

**Patient-based Prediction Algorithm of Relapse After allo-HSCT for Acute Leukemia and its Usefulness in the Decision-making Process Using a Machine Learning Approach**

**Supplementary Table 1. Definitions of the** **refinement of the Disease Risk Index (rDRI) and cytogenetic risk were excerpted from reference 7; Armand P, et al. *Blood.* 2014; 123(23):3664-3671.**

| Disease | Stage | rDRI Group |
| --- | --- | --- |
| AML favorable cytogenetic CR |  | LOW |
| AML intermediate cytogenetic CR |  | INT |
| ALL CR1 |  | INT |
| AML favorable cytogenetics | Advanced* | HI |
| ALL CR2 |  | HI |
| AML adverse cytogenetic CR |  | HI |
| ALL CR3 |  | HI |
| AML intermediate cytogenetics | Advanced* | HI |
| ALL | Advanced* | VH |
| AML adverse cytogenetics | Advanced* | VH |

**In AML, t(8;21), inv(16), and t(15;17) were classiﬁed as favorable, complex karyotype (4 or more abnormalities) as adverse, and all others as intermediate. In patients with ALL, cytogenetics were classiﬁed into 3 groups: Philadelphia chromosome–positive (Ph1; deﬁned by t(9;22) in a karyotype analysis or BCR-ABL translocation by ﬂuorescence *in situ* hybridization, where available); t(4;11); and all others.**

**CR; complete remission, CR1; first CR, CR2; second CR, CR3; third CR, INT; intermediate, HI; high, VH; very high**

***Advanced stage refers to induction failure or active relapse.**

**Supplementary Table 2. Summary of analysis of the number of nodes**

|  |  |  |  | In relapse | | | |
| --- | --- | --- | --- | --- | --- | --- | --- |
| Number of nodes | κ-statistic | Accuracy | AUC | TPR | TNR | FPR | FNR |
| 5 | 0.467 | 75.7% | 0.728 | 0.667 | 0.804 | 0.196 | 0.333 |
| 6 | 0.499 | 77.7% | 0.731 | 0.647 | 0.845 | 0.155 | 0.335 |
| **7** | **0.508** | **78.4%** | **0.746** | 0.627 | **0.866** | **0.134** | 0.373 |
| 8 | 0.490 | 77.7% | 0.76 | 0.608 | 0.866 | 0.134 | 0.396 |
| 9 | 0.446 | 75.7% | 0.74 | 0.588 | 0.845 | 0.155 | 0.412 |
| 10 | 0.459 | 76.4% | 0.744 | 0.588 | 85.6 | 0.144 | 0.412 |
| 11 | 0.446 | 75.7% | 0.739 | 0.588 | 0.845 | 0.155 | 0.412 |

The tree was analyzed the number of nodes between 6 and 11 and we adopted the number of nodes showing the highest κ-statistic value. When the number of nodes was seven, the κ-statistic value, accuracy and true negative rate (TNR, also called specificity) showed the highest scores and the false positive rate (FPR) of the relapse prediction was the lowest. We subsequently set the number of nodes to seven in the ADTree analysis.

AUC; area under the curve, Accuracy = (true positive + true negative) / all, TPR; true positive rate = true positive / (true positive + false negative) = 1-FNR, FNR; false negative rate = false negative / (false negative + true positive) = 1-TPR, TNR; true negative rate = true negative / (true negative + false positive) = 1- FPR, FPR; false positive rate = false positive / (false positive + true negative) = 1-TNR

**Supplementary Table 3. Detailed information on non-relapse mortality (NRM)**

| Cause of death | n |
| --- | --- |
| Infection, including bacterial, fungal, and viral infections | 7 |
| Respiratory failure, such as acute respiratory distress syndrome | 6 |
| Multi-organ failure | 6 |
| Interstitial pneumonia | 3 |
| Hemorrhage | 3 |
| Hepatic veno-occlusive disease | 3 |
| Graft-versus-host disease | 2 |
| Secondary malignancy | 2 |
| Renal disfunction | 2 |
| Liver dysfunction | 2 |
| Cerebral vascular disease | 2 |
| Heart attack | 1 |
| Unknown | 3 |
| Total | n=42 |

NRM was 19.4% (n=42). The most common cause of NRM was infection.
